# 2-Meth­oxy-*N*-[(*S*)-3-methyl­butan-2-yl]-6-{[(*S*)-3-methyl­butan-2-yl]amino}-3,5-dinitro­benzamide

**DOI:** 10.1107/S1600536811049749

**Published:** 2011-11-30

**Authors:** Xuefen Wu, Xi Chen, Yimin Hou

**Affiliations:** a College of Pharmacy, Henan University of Traditional Chinese Medicine, Zhengzhou, Henan 450008, People’s Republic of China; b School of Civil Engineering and Communication, North China University of Water Source and Electric Power, Zhengzhou 450011, People’s Republic of China

## Abstract

The title compound, C_18_H_28_N_4_O_6_, crystallizes with two mol­ecules in the asymmetric unit which differ slightly in conformation. The dihedral angle between the amide plane and the benzene ring are 72.6 (2) and 66.8 (2)° in the two mol­ecules. A strong intra­molecular N—H⋯O hydrogen bond between the amino and nitro groups occurs in each mol­ecule. The crystal structure features two symmetry-independent polymeric chains along [010] generated by N—H⋯O hydrogen bonds between the amide groups.

## Related literature

For aromatic mol­ecules with amide, nitro and alk­oxy groups and their use in medicinal chemistry, see: Neft & Farley (1971 ▶); Sykes *et al.* (1999 ▶).
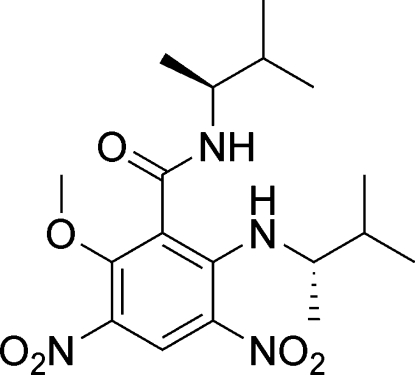


## Experimental

### 

#### Crystal data

C_18_H_28_N_4_O_6_
*M* *_r_* = 396.44
Monoclinic, 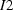

*a* = 21.1662 (16) Å
*b* = 9.8317 (7) Å
*c* = 22.565 (2) Å
β = 117.163 (1)°
*V* = 4177.8 (6) Å^3^
*Z* = 8
Mo *K*α radiation
μ = 0.10 mm^−1^
*T* = 296 K
0.68 × 0.22 × 0.10 mm

#### Data collection

Bruker APEXII CCD area-detector diffractometer
Absorption correction: multi-scan (*SADABS*; Sheldrick, 1996 ▶) *T*_min_ = 0.938, *T*_max_ = 0.990
12447 measured reflections
5062 independent reflections
2956 reflections with *I* > 2σ(*I*)
*R*_int_ = 0.031

#### Refinement

*R*[*F* ^2^ > 2σ(*F* ^2^)] = 0.060
*wR*(*F* ^2^) = 0.188
*S* = 0.99
5062 reflections
505 parameters
9 restraints
H-atom parameters constrained
Δρ_max_ = 0.62 e Å^−3^
Δρ_min_ = −0.23 e Å^−3^

### 

Data collection: *APEX2* (Bruker, 2007 ▶); cell refinement: *SAINT* (Bruker, 2007 ▶); data reduction: *SAINT*; program(s) used to solve structure: *SHELXTL* (Sheldrick, 2008 ▶); program(s) used to refine structure: *SHELXTL*; molecular graphics: *SHELXTL* (Sheldrick, 2008 ▶); software used to prepare material for publication: *SHELXTL*.

## Supplementary Material

Crystal structure: contains datablock(s) I, global. DOI: 10.1107/S1600536811049749/gk2433sup1.cif

Structure factors: contains datablock(s) I. DOI: 10.1107/S1600536811049749/gk2433Isup2.hkl

Supplementary material file. DOI: 10.1107/S1600536811049749/gk2433Isup3.cml

Additional supplementary materials:  crystallographic information; 3D view; checkCIF report

## Figures and Tables

**Table 1:** Hydrogen-bond geometry (Å, °)

*D*—H⋯*A*	*D*—H	H⋯*A*	*D*⋯*A*	*D*—H⋯*A*
N3—H3*A*⋯O7^i^	0.86	2.12	2.967 (4)	168
N6—H6*A*⋯O14^ii^	0.86	2.05	2.906 (4)	177
N7—H7*B*⋯O3	0.86	1.99	2.619 (6)	129
N8—H8*D*⋯O11	0.86	2.08	2.654 (5)	123

Symmetry codes: (i) 
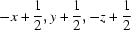
; (ii) 
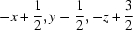
.

**Fig. 1.**
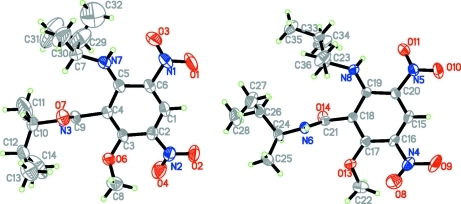
A view of the molecular strcuture of the title compound; the displacement ellipsoids drawn at the 30% probability level.

**Fig. 2.**
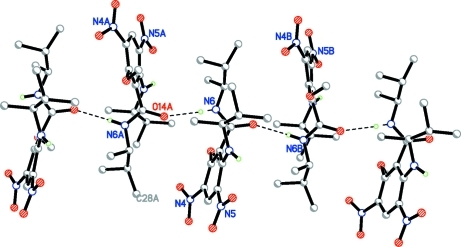
A view of the hydrogen bonded infinite zigzag chain in the [010] direction. The hydrogen bonds are shown as dashed lines and C-bound H atoms have been omitted for clarity.

**Fig. 3.**
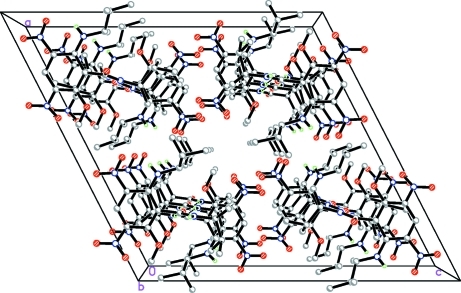
A view of the crystal packing of the title compound. Hydrogen bonds are shown as dashed lines; C-bound H atoms have been omitted for clarity.

## supplementary crystallographic information

### Comment

Amide, nitro and alkoxy groups exist in many active compounds, and have been shown to affect biological activity of compounds in varying degrees (Neft & Farley, 1971; Sykes *et al.*, 1999). We synthesised the title compound and plan to examine its function as potential drug or as a prodrug.

The dihedral angle between the amide plane and the benzene ring in the two molecules are 72.6 (2)° and 66.8 (2)°, respectively. The molecules are linked by intermolecular N—H···O hydrogen bonding, forming one-dimensional infinite zigzag chain in the [010] directions, in which the amide H atom acts as a donor and the carbonyl group act as acceptor (Fig.2).

### Experimental

To a solution of (*S*)-3-methylbutan-2-amine (0.45 g, 5 mmol)) in dry dichloromethane (30 ml) and triethylamine (1 mL) was added 2,6-dimethoxy-3,5-dinitrobenzoyl chloride (0.58 g, 2 mmol) at 0°C. The mixture was stirred at room temperature for another 2 h. The residue was subjected to chromatography (petroleum ether/acetone, 5:1) to provide the product as a yellow solid (80.8 mg, 10.2%). The crystal of the title compound was grown from ethyl acetate.

### Refinement

The C- and N-bound H-atoms were included in calculated positions and treated as riding atoms with C-H = 0.93, 0.96 and 0.98Å for CH(aromatic), CH_3_ and CH(methine) H atoms, respectively and N-H =0.86Å, with U_iso_(H)= k U_eq_(C,N), where k = 1.5 for CH_3_ H atoms and k = 1.2 for all other H atoms. The highest residual peak in the final electron-density difference map is located close to a strongly vibrating alkyl substituent. Due to negligible anomalous dispersion effect Friedel pairs were merged. The absolute structure was determined relative to the known chiral centers.

### Crystal data

**Table:**

C_18_H_28_N_4_O_6_	*F*(000) = 1696
*M**_r_* = 396.44	*D*_x_ = 1.261 Mg m^−^^3^
Monoclinic, *I*2	Melting point: 426 K
Hall symbol: I 2y	Mo *K*α radiation, λ = 0.71073 Å
*a* = 21.1662 (16) Å	Cell parameters from 12448 reflections
*b* = 9.8317 (7) Å	θ = 1.0–27.7°
*c* = 22.565 (2) Å	µ = 0.10 mm^−^^1^
β = 117.163 (1)°	*T* = 296 K
*V* = 4177.8 (6) Å^3^	Block, colourless
*Z* = 8	0.68 × 0.22 × 0.10 mm

### Data collection

**Table:**

Bruker APEXII CCD area-detector diffractometer	5062 independent reflections
Radiation source: fine-focus sealed tube	2956 reflections with *I* > 2σ(*I*)
graphite	*R*_int_ = 0.031
phi and ω scans	θ_max_ = 27.7°, θ_min_ = 1.8°
Absorption correction: multi-scan (*SADABS*; Sheldrick, 1996)	*h* = −27→27
*T*_min_ = 0.938, *T*_max_ = 0.990	*k* = −10→12
12447 measured reflections	*l* = −29→28

### Refinement

**Table:**

Refinement on *F*^2^	Primary atom site location: structure-invariant direct methods
Least-squares matrix: full	Secondary atom site location: difference Fourier map
*R*[*F*^2^ > 2σ(*F*^2^)] = 0.060	Hydrogen site location: inferred from neighbouring sites
*wR*(*F*^2^) = 0.188	H-atom parameters constrained
*S* = 0.99	*w* = 1/[σ^2^(*F*_o_^2^) + (0.110*P*)^2^] where *P* = (*F*_o_^2^ + 2*F*_c_^2^)/3
5062 reflections	(Δ/σ)_max_ = 0.003
505 parameters	Δρ_max_ = 0.62 e Å^−^^3^
9 restraints	Δρ_min_ = −0.23 e Å^−^^3^

### Special details

**Table:**

Geometry. All e.s.d.'s (except the e.s.d. in the dihedral angle between two l.s. planes) are estimated using the full covariance matrix. The cell e.s.d.'s are taken into account individually in the estimation of e.s.d.'s in distances, angles and torsion angles; correlations between e.s.d.'s in cell parameters are only used when they are defined by crystal symmetry. An approximate (isotropic) treatment of cell e.s.d.'s is used for estimating e.s.d.'s involving l.s. planes.
Refinement. Refinement of *F*^2^ against ALL reflections. The weighted *R*-factor *wR* and goodness of fit *S* are based on *F*^2^, conventional *R*-factors *R* are based on *F*, with *F* set to zero for negative *F*^2^. The threshold expression of *F*^2^ > σ(*F*^2^) is used only for calculating *R*-factors(gt) *etc*. and is not relevant to the choice of reflections for refinement. *R*-factors based on *F*^2^ are statistically about twice as large as those based on *F*, and *R*- factors based on ALL data will be even larger.

### Fractional atomic coordinates and isotropic or equivalent isotropic displacement parameters (Å^2^)

**Table:**

	*x*	*y*	*z*	*U*_iso_*/*U*_eq_	
C1	0.2149 (3)	0.0458 (5)	0.4202 (2)	0.0657 (13)	
H1A	0.2120	0.0726	0.4584	0.079*	
C2	0.2775 (3)	0.0626 (5)	0.4176 (2)	0.0601 (12)	
C3	0.2829 (3)	0.0145 (5)	0.3604 (2)	0.0536 (11)	
C4	0.2239 (2)	−0.0351 (4)	0.3071 (2)	0.0496 (10)	
C5	0.1559 (3)	−0.0464 (5)	0.3066 (2)	0.0559 (11)	
C6	0.1564 (3)	−0.0089 (5)	0.3689 (2)	0.0606 (12)	
C7	0.0754 (3)	−0.0949 (9)	0.1834 (3)	0.094 (2)	
H7A	0.1180	−0.0939	0.1766	0.113*	
C8	0.4036 (3)	−0.0608 (9)	0.4051 (3)	0.104 (2)	
H8A	0.4435	−0.0541	0.3958	0.156*	
H8B	0.3907	−0.1546	0.4044	0.156*	
H8C	0.4161	−0.0231	0.4483	0.156*	
C9	0.2352 (2)	−0.0956 (5)	0.2504 (2)	0.0508 (10)	
C10	0.2536 (3)	−0.0434 (6)	0.1525 (2)	0.0695 (14)	
H10A	0.2298	−0.1309	0.1358	0.083*	
C11	0.2157 (6)	0.0619 (14)	0.0979 (4)	0.176 (6)	
H11A	0.1670	0.0688	0.0891	0.264*	
H11B	0.2179	0.0345	0.0580	0.264*	
H11C	0.2385	0.1486	0.1122	0.264*	
C12	0.3296 (4)	−0.0607 (8)	0.1670 (4)	0.095 (2)	
H12A	0.3301	−0.0869	0.1253	0.114*	
C13	0.3716 (5)	−0.1605 (11)	0.2172 (4)	0.129 (3)	
H13A	0.3493	−0.2481	0.2047	0.193*	
H13B	0.3747	−0.1344	0.2594	0.193*	
H13C	0.4184	−0.1650	0.2205	0.193*	
C14	0.3696 (7)	0.0804 (12)	0.1895 (8)	0.208 (7)	
H14A	0.4182	0.0694	0.1985	0.313*	
H14B	0.3678	0.1113	0.2291	0.313*	
H14C	0.3470	0.1461	0.1546	0.313*	
C15	0.2353 (2)	0.3234 (5)	0.93893 (19)	0.0521 (10)	
H15A	0.2345	0.3099	0.9794	0.063*	
C16	0.2961 (2)	0.3005 (4)	0.93438 (19)	0.0490 (10)	
C17	0.2997 (2)	0.3289 (4)	0.87464 (18)	0.0467 (9)	
C18	0.23767 (19)	0.3671 (4)	0.81838 (17)	0.0394 (8)	
C19	0.17142 (19)	0.3801 (4)	0.82059 (17)	0.0398 (8)	
C20	0.1747 (2)	0.3663 (5)	0.8847 (2)	0.0497 (10)	
C21	0.24722 (19)	0.4191 (4)	0.76034 (19)	0.0399 (9)	
C22	0.4217 (3)	0.3907 (7)	0.9148 (3)	0.0807 (16)	
H22A	0.4590	0.3786	0.9023	0.121*	
H22B	0.4357	0.3512	0.9579	0.121*	
H22C	0.4128	0.4860	0.9164	0.121*	
C23	0.0912 (2)	0.3596 (5)	0.69793 (18)	0.0498 (10)	
H23A	0.1288	0.3864	0.6864	0.060*	
C24	0.2849 (2)	0.3677 (5)	0.6750 (2)	0.0521 (10)	
H24A	0.3017	0.4620	0.6828	0.063*	
C25	0.3471 (3)	0.2764 (7)	0.6830 (3)	0.0794 (16)	
H25A	0.3855	0.2871	0.7270	0.119*	
H25B	0.3628	0.3018	0.6507	0.119*	
H25C	0.3320	0.1831	0.6762	0.119*	
C26	0.2207 (3)	0.3596 (6)	0.6053 (2)	0.0685 (14)	
H26A	0.1826	0.4139	0.6069	0.082*	
C27	0.1923 (4)	0.2182 (8)	0.5859 (3)	0.097 (2)	
H27A	0.1819	0.1803	0.6196	0.145*	
H27B	0.2272	0.1629	0.5812	0.145*	
H27C	0.1498	0.2208	0.5443	0.145*	
C28	0.2375 (4)	0.4239 (7)	0.5523 (3)	0.100 (2)	
H28A	0.1968	0.4163	0.5094	0.150*	
H28B	0.2770	0.3776	0.5515	0.150*	
H28C	0.2491	0.5182	0.5627	0.150*	
N1	0.0926 (3)	−0.0156 (6)	0.3776 (3)	0.0814 (13)	
N2	0.3328 (3)	0.1353 (5)	0.4711 (2)	0.0753 (12)	
N3	0.2437 (2)	−0.0073 (4)	0.21059 (17)	0.0564 (9)	
H3A	0.2435	0.0777	0.2195	0.068*	
N4	0.3536 (2)	0.2302 (5)	0.99008 (18)	0.0655 (11)	
N5	0.1125 (2)	0.3863 (5)	0.89616 (19)	0.0641 (11)	
N6	0.26495 (17)	0.3299 (3)	0.72681 (15)	0.0440 (7)	
H6A	0.2648	0.2450	0.7360	0.053*	
O1	0.0952 (3)	0.0318 (6)	0.4284 (3)	0.1188 (18)	
O2	0.3313 (2)	0.1448 (5)	0.5239 (2)	0.0986 (14)	
O3	0.0388 (2)	−0.0701 (6)	0.3351 (2)	0.1003 (15)	
O4	0.3773 (3)	0.1932 (6)	0.4594 (2)	0.1203 (19)	
N7	0.0976 (2)	−0.0935 (5)	0.2554 (2)	0.0702 (11)	
H7B	0.0675	−0.1296	0.2664	0.084*	
O6	0.34459 (17)	0.0131 (4)	0.35551 (15)	0.0648 (9)	
O7	0.2352 (2)	−0.2202 (3)	0.24356 (19)	0.0683 (9)	
O8	0.3963 (2)	0.1660 (5)	0.9792 (2)	0.1004 (14)	
O9	0.3522 (2)	0.2307 (5)	1.04399 (16)	0.0983 (14)	
O10	0.1161 (2)	0.3519 (5)	0.94979 (17)	0.0871 (12)	
O11	0.05903 (19)	0.4372 (5)	0.85230 (18)	0.0852 (13)	
N8	0.11081 (17)	0.4067 (4)	0.76599 (15)	0.0479 (8)	
H8D	0.0803	0.4564	0.7712	0.058*	
O13	0.35904 (14)	0.3261 (4)	0.86735 (14)	0.0668 (10)	
O14	0.23905 (17)	0.5416 (3)	0.74670 (16)	0.0528 (7)	
C33	0.0220 (2)	0.4323 (5)	0.6502 (2)	0.0568 (12)	
H33A	−0.0146	0.4086	0.6635	0.068*	
C34	0.0310 (3)	0.5837 (7)	0.6548 (4)	0.108 (2)	
H34A	0.0465	0.6121	0.7000	0.162*	
H34B	−0.0135	0.6264	0.6266	0.162*	
H34C	0.0658	0.6097	0.6407	0.162*	
C35	−0.0028 (3)	0.3829 (10)	0.5789 (2)	0.108 (2)	
H35A	−0.0462	0.4281	0.5499	0.162*	
H35B	−0.0107	0.2865	0.5768	0.162*	
H35C	0.0329	0.4030	0.5651	0.162*	
C36	0.0854 (3)	0.2077 (6)	0.6950 (3)	0.0895 (18)	
H36A	0.1302	0.1687	0.7254	0.134*	
H36B	0.0730	0.1774	0.6506	0.134*	
H36C	0.0495	0.1796	0.7072	0.134*	
C29	0.0361 (5)	−0.2255 (10)	0.1558 (4)	0.142 (3)	
H29A	0.0674	−0.3010	0.1761	0.212*	
H29B	0.0196	−0.2278	0.1085	0.212*	
H29C	−0.0036	−0.2311	0.1653	0.212*	
C30	0.0320 (7)	0.0370 (14)	0.1527 (5)	0.167 (5)	
H30A	0.0607	0.1154	0.1769	0.200*	
C31	0.0144 (10)	0.051 (2)	0.0775 (5)	0.299 (11)	
H31A	−0.0137	0.1308	0.0591	0.448*	
H31B	−0.0118	−0.0280	0.0535	0.448*	
H31C	0.0577	0.0572	0.0739	0.448*	
C32	−0.0292 (8)	0.041 (2)	0.1559 (7)	0.230 (7)	
H32A	−0.0200	0.0389	0.2017	0.344*	
H32B	−0.0577	−0.0362	0.1330	0.344*	
H32C	−0.0542	0.1231	0.1354	0.344*	

### Atomic displacement parameters (Å^2^)

**Table:**

	*U*^11^	*U*^22^	*U*^33^	*U*^12^	*U*^13^	*U*^23^
C1	0.090 (4)	0.063 (3)	0.058 (3)	0.023 (3)	0.046 (3)	0.012 (2)
C2	0.074 (3)	0.054 (3)	0.053 (3)	0.007 (2)	0.029 (2)	0.006 (2)
C3	0.069 (3)	0.047 (2)	0.058 (3)	0.005 (2)	0.040 (2)	0.006 (2)
C4	0.061 (3)	0.043 (2)	0.052 (2)	0.0032 (19)	0.031 (2)	0.0044 (19)
C5	0.070 (3)	0.045 (2)	0.065 (3)	0.007 (2)	0.041 (3)	0.005 (2)
C6	0.073 (3)	0.060 (3)	0.065 (3)	0.011 (2)	0.046 (3)	0.008 (2)
C7	0.061 (3)	0.138 (6)	0.079 (4)	−0.009 (4)	0.028 (3)	−0.031 (4)
C8	0.082 (4)	0.133 (7)	0.099 (4)	0.036 (4)	0.044 (4)	0.033 (4)
C9	0.059 (3)	0.045 (3)	0.056 (2)	0.000 (2)	0.033 (2)	0.001 (2)
C10	0.085 (4)	0.074 (4)	0.056 (3)	0.000 (3)	0.038 (3)	0.001 (3)
C11	0.233 (11)	0.234 (14)	0.103 (6)	0.110 (11)	0.114 (7)	0.078 (8)
C12	0.106 (5)	0.102 (5)	0.100 (4)	0.006 (4)	0.067 (4)	−0.019 (4)
C13	0.139 (7)	0.127 (7)	0.150 (7)	−0.004 (6)	0.091 (6)	0.000 (6)
C14	0.228 (12)	0.121 (8)	0.39 (2)	−0.074 (8)	0.240 (14)	−0.105 (11)
C15	0.064 (3)	0.054 (3)	0.0350 (19)	−0.007 (2)	0.0199 (19)	−0.0027 (19)
C16	0.054 (2)	0.045 (2)	0.0362 (19)	0.0025 (18)	0.0102 (18)	0.0007 (18)
C17	0.050 (2)	0.044 (2)	0.042 (2)	0.0044 (18)	0.0171 (19)	−0.0043 (18)
C18	0.0428 (19)	0.036 (2)	0.0350 (17)	0.0004 (16)	0.0135 (16)	−0.0025 (16)
C19	0.042 (2)	0.039 (2)	0.0390 (19)	−0.0017 (16)	0.0183 (17)	−0.0023 (16)
C20	0.052 (2)	0.053 (3)	0.049 (2)	−0.003 (2)	0.028 (2)	−0.002 (2)
C21	0.0393 (19)	0.035 (2)	0.043 (2)	−0.0028 (16)	0.0161 (16)	−0.0018 (17)
C22	0.060 (3)	0.079 (4)	0.096 (4)	−0.003 (3)	0.029 (3)	0.002 (3)
C23	0.044 (2)	0.063 (3)	0.0373 (19)	0.001 (2)	0.0141 (17)	0.003 (2)
C24	0.065 (3)	0.048 (2)	0.056 (2)	0.001 (2)	0.039 (2)	0.004 (2)
C25	0.071 (3)	0.100 (4)	0.081 (3)	0.018 (3)	0.047 (3)	0.015 (3)
C26	0.080 (3)	0.077 (4)	0.056 (3)	0.023 (3)	0.037 (2)	0.016 (3)
C27	0.113 (5)	0.106 (5)	0.063 (3)	−0.019 (4)	0.034 (3)	−0.015 (4)
C28	0.145 (6)	0.095 (5)	0.086 (4)	0.044 (4)	0.074 (4)	0.036 (4)
N1	0.085 (3)	0.089 (3)	0.098 (4)	0.015 (3)	0.066 (3)	0.013 (3)
N2	0.087 (3)	0.075 (3)	0.060 (3)	0.006 (3)	0.030 (2)	−0.005 (2)
N3	0.076 (3)	0.047 (2)	0.054 (2)	0.0021 (18)	0.036 (2)	−0.0006 (18)
N4	0.071 (3)	0.066 (3)	0.044 (2)	−0.002 (2)	0.0124 (19)	0.008 (2)
N5	0.063 (2)	0.083 (3)	0.057 (2)	−0.013 (2)	0.036 (2)	−0.014 (2)
N6	0.0568 (19)	0.0363 (17)	0.0436 (16)	0.0000 (15)	0.0270 (15)	0.0033 (15)
O1	0.127 (4)	0.154 (5)	0.125 (4)	0.016 (4)	0.101 (3)	−0.010 (4)
O2	0.119 (3)	0.115 (4)	0.064 (2)	0.008 (3)	0.044 (2)	−0.024 (2)
O3	0.075 (3)	0.140 (4)	0.106 (3)	0.001 (3)	0.058 (3)	0.005 (3)
O4	0.126 (4)	0.139 (5)	0.093 (3)	−0.054 (4)	0.047 (3)	−0.039 (3)
N7	0.062 (2)	0.081 (3)	0.076 (3)	−0.008 (2)	0.039 (2)	−0.008 (2)
O6	0.0598 (19)	0.080 (2)	0.0599 (18)	−0.0005 (16)	0.0322 (16)	0.0067 (17)
O7	0.091 (3)	0.0421 (18)	0.084 (2)	0.0023 (16)	0.050 (2)	−0.0027 (17)
O8	0.089 (3)	0.115 (4)	0.091 (3)	0.049 (3)	0.036 (2)	0.045 (3)
O9	0.115 (3)	0.113 (4)	0.0389 (18)	0.021 (3)	0.0106 (19)	0.014 (2)
O10	0.096 (3)	0.122 (3)	0.066 (2)	−0.016 (3)	0.0562 (19)	−0.003 (2)
O11	0.059 (2)	0.135 (4)	0.071 (2)	0.018 (2)	0.038 (2)	−0.002 (2)
N8	0.0408 (17)	0.058 (2)	0.0414 (17)	0.0061 (16)	0.0153 (14)	−0.0012 (17)
O13	0.0403 (16)	0.103 (3)	0.0490 (15)	0.0133 (17)	0.0134 (13)	−0.0031 (18)
O14	0.075 (2)	0.0330 (16)	0.0576 (16)	−0.0021 (14)	0.0363 (15)	−0.0008 (13)
C33	0.040 (2)	0.078 (3)	0.044 (2)	−0.001 (2)	0.0121 (18)	0.011 (2)
C34	0.086 (4)	0.080 (4)	0.106 (5)	0.011 (3)	−0.002 (4)	0.026 (4)
C35	0.080 (4)	0.164 (8)	0.051 (3)	0.021 (5)	0.004 (3)	0.006 (4)
C36	0.095 (4)	0.068 (4)	0.073 (4)	0.007 (3)	0.010 (3)	−0.011 (3)
C29	0.145 (7)	0.101 (6)	0.128 (7)	0.002 (5)	0.017 (6)	−0.042 (5)
C30	0.191 (11)	0.158 (10)	0.096 (6)	−0.038 (9)	0.017 (7)	0.036 (6)
C31	0.41 (2)	0.30 (3)	0.098 (7)	−0.07 (2)	0.037 (11)	0.009 (11)
C32	0.253 (10)	0.196 (10)	0.224 (10)	0.044 (9)	0.095 (8)	0.049 (8)

### Geometric parameters (Å, °)

**Table:**

C1—C6	1.361 (7)	C23—N8	1.471 (5)
C1—C2	1.364 (7)	C23—C36	1.497 (7)
C1—H1A	0.9300	C23—C33	1.541 (6)
C2—C3	1.428 (6)	C23—H23A	0.9800
C2—N2	1.430 (7)	C24—N6	1.461 (5)
C3—O6	1.359 (5)	C24—C25	1.536 (7)
C3—C4	1.368 (6)	C24—C26	1.541 (7)
C4—C5	1.440 (6)	C24—H24A	0.9800
C4—C9	1.525 (6)	C25—H25A	0.9600
C5—N7	1.330 (6)	C25—H25B	0.9600
C5—C6	1.447 (6)	C25—H25C	0.9600
C6—N1	1.453 (6)	C26—C27	1.498 (9)
C7—N7	1.472 (7)	C26—C28	1.533 (7)
C7—C29	1.501 (12)	C26—H26A	0.9800
C7—C30	1.557 (15)	C27—H27A	0.9600
C7—H7A	0.9800	C27—H27B	0.9600
C8—O6	1.437 (7)	C27—H27C	0.9600
C8—H8A	0.9600	C28—H28A	0.9600
C8—H8B	0.9600	C28—H28B	0.9600
C8—H8C	0.9600	C28—H28C	0.9600
C9—O7	1.236 (6)	N1—O1	1.215 (6)
C9—N3	1.320 (6)	N1—O3	1.228 (7)
C10—N3	1.463 (6)	N2—O2	1.212 (5)
C10—C12	1.498 (8)	N2—O4	1.226 (6)
C10—C11	1.526 (10)	N3—H3A	0.8600
C10—H10A	0.9800	N4—O8	1.215 (6)
C11—H11A	0.9600	N4—O9	1.231 (5)
C11—H11B	0.9600	N5—O11	1.219 (5)
C11—H11C	0.9600	N5—O10	1.225 (5)
C12—C13	1.454 (11)	N6—H6A	0.8600
C12—C14	1.583 (12)	N7—H7B	0.8600
C12—H12A	0.9800	N8—H8D	0.8600
C13—H13A	0.9600	C33—C34	1.499 (9)
C13—H13B	0.9600	C33—C35	1.526 (7)
C13—H13C	0.9600	C33—H33A	0.9800
C14—H14A	0.9600	C34—H34A	0.9600
C14—H14B	0.9600	C34—H34B	0.9600
C14—H14C	0.9600	C34—H34C	0.9600
C15—C16	1.357 (6)	C35—H35A	0.9600
C15—C20	1.373 (6)	C35—H35B	0.9600
C15—H15A	0.9300	C35—H35C	0.9600
C16—C17	1.412 (5)	C36—H36A	0.9600
C16—N4	1.463 (6)	C36—H36B	0.9600
C17—O13	1.340 (5)	C36—H36C	0.9600
C17—C18	1.398 (5)	C29—H29A	0.9600
C18—C19	1.432 (5)	C29—H29B	0.9600
C18—C21	1.503 (5)	C29—H29C	0.9600
C19—N8	1.337 (5)	C30—C32	1.332 (16)
C19—C20	1.422 (5)	C30—C31	1.567 (15)
C20—N5	1.468 (5)	C30—H30A	0.9800
C21—O14	1.236 (5)	C31—H31A	0.9600
C21—N6	1.320 (5)	C31—H31B	0.9600
C22—O13	1.419 (6)	C31—H31C	0.9600
C22—H22A	0.9600	C32—H32A	0.9600
C22—H22B	0.9600	C32—H32B	0.9600
C22—H22C	0.9600	C32—H32C	0.9600
			
C6—C1—C2	122.4 (4)	C25—C24—H24A	108.1
C6—C1—H1A	118.8	C26—C24—H24A	108.1
C2—C1—H1A	118.8	C24—C25—H25A	109.5
C1—C2—C3	118.6 (5)	C24—C25—H25B	109.5
C1—C2—N2	117.5 (4)	H25A—C25—H25B	109.5
C3—C2—N2	123.8 (5)	C24—C25—H25C	109.5
O6—C3—C4	116.8 (4)	H25A—C25—H25C	109.5
O6—C3—C2	123.5 (4)	H25B—C25—H25C	109.5
C4—C3—C2	119.6 (4)	C27—C26—C28	111.0 (5)
C3—C4—C5	122.9 (4)	C27—C26—C24	113.3 (4)
C3—C4—C9	116.5 (4)	C28—C26—C24	111.3 (5)
C5—C4—C9	120.2 (4)	C27—C26—H26A	106.9
N7—C5—C4	124.1 (4)	C28—C26—H26A	106.9
N7—C5—C6	121.7 (4)	C24—C26—H26A	106.9
C4—C5—C6	114.1 (4)	C26—C27—H27A	109.5
C1—C6—C5	121.9 (4)	C26—C27—H27B	109.5
C1—C6—N1	116.3 (4)	H27A—C27—H27B	109.5
C5—C6—N1	121.6 (5)	C26—C27—H27C	109.5
N7—C7—C29	107.6 (7)	H27A—C27—H27C	109.5
N7—C7—C30	108.0 (7)	H27B—C27—H27C	109.5
C29—C7—C30	115.2 (6)	C26—C28—H28A	109.5
N7—C7—H7A	108.6	C26—C28—H28B	109.5
C29—C7—H7A	108.6	H28A—C28—H28B	109.5
C30—C7—H7A	108.6	C26—C28—H28C	109.5
O6—C8—H8A	109.5	H28A—C28—H28C	109.5
O6—C8—H8B	109.5	H28B—C28—H28C	109.5
H8A—C8—H8B	109.5	O1—N1—O3	122.1 (5)
O6—C8—H8C	109.5	O1—N1—C6	117.8 (6)
H8A—C8—H8C	109.5	O3—N1—C6	120.1 (5)
H8B—C8—H8C	109.5	O2—N2—O4	122.8 (5)
O7—C9—N3	124.0 (4)	O2—N2—C2	119.3 (5)
O7—C9—C4	120.1 (4)	O4—N2—C2	117.6 (4)
N3—C9—C4	115.9 (4)	C9—N3—C10	124.8 (4)
N3—C10—C12	114.2 (5)	C9—N3—H3A	117.6
N3—C10—C11	108.7 (5)	C10—N3—H3A	117.6
C12—C10—C11	111.7 (6)	O8—N4—O9	123.9 (4)
N3—C10—H10A	107.3	O8—N4—C16	118.4 (4)
C12—C10—H10A	107.3	O9—N4—C16	117.4 (4)
C11—C10—H10A	107.3	O11—N5—O10	122.1 (4)
C10—C11—H11A	109.5	O11—N5—C20	119.1 (4)
C10—C11—H11B	109.5	O10—N5—C20	118.8 (4)
H11A—C11—H11B	109.5	C21—N6—C24	123.5 (3)
C10—C11—H11C	109.5	C21—N6—H6A	118.3
H11A—C11—H11C	109.5	C24—N6—H6A	118.3
H11B—C11—H11C	109.5	C5—N7—C7	131.7 (4)
C13—C12—C10	117.4 (6)	C5—N7—H7B	114.2
C13—C12—C14	107.2 (8)	C7—N7—H7B	114.2
C10—C12—C14	109.5 (6)	C3—O6—C8	117.9 (4)
C13—C12—H12A	107.4	C19—N8—C23	126.5 (3)
C10—C12—H12A	107.4	C19—N8—H8D	116.7
C14—C12—H12A	107.4	C23—N8—H8D	116.7
C12—C13—H13A	109.5	C17—O13—C22	120.3 (4)
C12—C13—H13B	109.5	C34—C33—C35	111.1 (6)
H13A—C13—H13B	109.5	C34—C33—C23	111.3 (4)
C12—C13—H13C	109.5	C35—C33—C23	110.5 (4)
H13A—C13—H13C	109.5	C34—C33—H33A	107.9
H13B—C13—H13C	109.5	C35—C33—H33A	107.9
C12—C14—H14A	109.5	C23—C33—H33A	107.9
C12—C14—H14B	109.5	C33—C34—H34A	109.5
H14A—C14—H14B	109.5	C33—C34—H34B	109.5
C12—C14—H14C	109.5	H34A—C34—H34B	109.5
H14A—C14—H14C	109.5	C33—C34—H34C	109.5
H14B—C14—H14C	109.5	H34A—C34—H34C	109.5
C16—C15—C20	121.0 (4)	H34B—C34—H34C	109.5
C16—C15—H15A	119.5	C33—C35—H35A	109.5
C20—C15—H15A	119.5	C33—C35—H35B	109.5
C15—C16—C17	120.4 (4)	H35A—C35—H35B	109.5
C15—C16—N4	117.4 (4)	C33—C35—H35C	109.5
C17—C16—N4	121.8 (4)	H35A—C35—H35C	109.5
O13—C17—C18	116.0 (3)	H35B—C35—H35C	109.5
O13—C17—C16	125.1 (3)	C23—C36—H36A	109.5
C18—C17—C16	118.9 (4)	C23—C36—H36B	109.5
C17—C18—C19	121.4 (3)	H36A—C36—H36B	109.5
C17—C18—C21	116.0 (3)	C23—C36—H36C	109.5
C19—C18—C21	121.6 (3)	H36A—C36—H36C	109.5
N8—C19—C20	122.5 (3)	H36B—C36—H36C	109.5
N8—C19—C18	121.9 (3)	C7—C29—H29A	109.5
C20—C19—C18	115.6 (3)	C7—C29—H29B	109.5
C15—C20—C19	121.9 (4)	H29A—C29—H29B	109.5
C15—C20—N5	115.5 (4)	C7—C29—H29C	109.5
C19—C20—N5	122.4 (4)	H29A—C29—H29C	109.5
O14—C21—N6	123.3 (4)	H29B—C29—H29C	109.5
O14—C21—C18	119.3 (3)	C32—C30—C7	113.0 (12)
N6—C21—C18	117.4 (3)	C32—C30—C31	107.5 (13)
O13—C22—H22A	109.5	C7—C30—C31	110.7 (13)
O13—C22—H22B	109.5	C32—C30—H30A	108.5
H22A—C22—H22B	109.5	C7—C30—H30A	108.5
O13—C22—H22C	109.5	C31—C30—H30A	108.5
H22A—C22—H22C	109.5	C30—C31—H31A	109.5
H22B—C22—H22C	109.5	C30—C31—H31B	109.5
N8—C23—C36	109.8 (4)	H31A—C31—H31B	109.5
N8—C23—C33	108.0 (3)	C30—C31—H31C	109.5
C36—C23—C33	113.6 (4)	H31A—C31—H31C	109.5
N8—C23—H23A	108.5	H31B—C31—H31C	109.5
C36—C23—H23A	108.5	C30—C32—H32A	109.5
C33—C23—H23A	108.5	C30—C32—H32B	109.5
N6—C24—C25	107.6 (4)	H32A—C32—H32B	109.5
N6—C24—C26	111.2 (3)	C30—C32—H32C	109.5
C25—C24—C26	113.6 (4)	H32A—C32—H32C	109.5
N6—C24—H24A	108.1	H32B—C32—H32C	109.5

### Hydrogen-bond geometry (Å, °)

**Table:**

*D*—H···*A*	*D*—H	H···*A*	*D*···*A*	*D*—H···*A*
N3—H3A···O7^i^	0.86	2.12	2.967 (4)	168
N6—H6A···O14^ii^	0.86	2.05	2.906 (4)	177
N7—H7B···O3	0.86	1.99	2.619 (6)	129
N8—H8D···O11	0.86	2.08	2.654 (5)	123

Symmetry codes: (i) −*x*+1/2, *y*+1/2, −*z*+1/2; (ii) −*x*+1/2, *y*−1/2, −*z*+3/2.
